# Cereulide Exposure Caused Cytopathogenic Damages of Liver and Kidney in Mice

**DOI:** 10.3390/ijms22179148

**Published:** 2021-08-24

**Authors:** Danyang Li, Ruqin Lin, Yangyang Xu, Qingmei Chen, Fengru Deng, Yiqun Deng, Jikai Wen

**Affiliations:** 1 Guangdong Provincial Key Laboratory of Protein Function and Regulation in Agricultural Organisms, College of Life Sciences, South China Agricultural University, Guangzhou 510642, China; lidanyang@stu.scau.edu.cn (D.L.); linruqin@scau.edu.cn (R.L.); xuyangyang@stu.scau.edu.cn (Y.X.); chenqingmei@scau.edu.cn (Q.C.); dengfengru@scau.edu.cn (F.D.); 2 Key Laboratory of Zoonosis of Ministry of Agriculture and Rural Affairs, South China Agricultural University, Guangzhou 510642, China; 3 Guangdong Laboratory for Lingnan Modern Agriculture, South China Agricultural University, Guangzhou 510642, China

**Keywords:** cereulide, endoplasmic reticulum stress, reactive oxygen species, apoptosis, sodium butyrate

## Abstract

Cereulide is one of the main food-borne toxins for vomiting synthesized by *Bacillus cereus*, and it widely contaminates meat, eggs, milk, and starchy foods. However, the toxicological effects and mechanisms of the long-time exposure of cereulide in vivo remain unknown. In this study, oral administration of 50 and 200 μg/kg body weight cereulide in the mice for 28 days caused oxidative stress in liver and kidney tissues and induce abnormal expression of inflammatory factors. In pathogenesis, cereulide exposure activated endoplasmic reticulum stress (ER stress) via the pathways of inositol-requiring enzyme 1α (IRE1α)/Xbox binding protein (XBP1) and PRKR-like ER kinase (PERK)/eukaryotic translation initiation factor 2α (eIF2α), and consequently led to the apoptosis and tissue damages in mouse liver and kidney. In vitro, we confirmed that the accumulation of reactive oxygen species (ROS) caused by cereulide is the main factor leading to ER stress in HepaRG and HEK293T cells. Supplementation of sodium butyrate (NaB) inhibited the activations of IRE1α/XBP1 and PERK/eIF2α pathways caused by cereulide exposure in mice, and reduced the cell apoptosis in liver and kidney. In conclusion, this study provides a new insight in understanding the toxicological mechanism and prevention of cereulide exposure.

## 1. Introduction

Cereulide is a cyclic dodecadepsipeptide toxin, which is synthesized by the food-borne pathogen *Bacillus cereus* (*B. cereus*) through a non-ribosomal peptide synthetase system [1]. In the nature of strong thermal stability and acid resistance, cereulide is difficult to remove from the food chain and detoxified in animal digestive tracts [2,3]. Cereulide food poisoning causes nausea, vomiting and discomfort in mild cases, and liver failure and acute encephalopathy in severe cases [4,5,6]. The part of cereulide ingested with food is rapidly excreted with feces while the part of cereulide toxin is absorbed, passed through intestinal barriers into the blood, and distributed throughout the body [7]. Cereulide and K^+^ ions form a stable complex, which destroys the electrochemical potential gradient on the lipid membrane and causes mitochondria to swell [8,9]. Studies have found that cereulide causes mitochondrial depolarization and decreases mitochondrial maximum respiration [10,11]. Cereulide exposure aggravates the accumulation of ROS, activates the mitochondrial stress pathway, and causes the release of cytochrome C into the cytoplasm, all of which contribute to cereulide-induced apoptosis [12].

The risk of cereulide in food poisoning events is possibly underestimated. Because cereulide poisoning is usually transient and relatively difficult to detect, it is often classified as food poisoning of unknown reasons and is ignored [13,14]. Current studies on the toxicity of cereulide are limited to toxic reactions under acute exposure. However, studies at the cellular level have found that long-term low-dose exposure to cereulide causes the loss of insulin secretion capacity of β cells [15]. In addition, prolonged exposure to cereulide at low dose increases the cytotoxic effects and immune responses in the human intestine caused by deoxynivalenol (DON) [16]. From the above in vitro studies, it can be inferred that long-time cereulide exposure at sub-emetic concentrations may cause risks to the body’s health. However, in vivo studies focusing on the long-time exposure of cereulide are still lacking, and its toxicological mechanisms are unclear in animals.

In this study, a mouse model of long-time cereulide exposure was constructed, and we found that cereulide exposure activates ER stress and promotes apoptosis of liver and kidney cells. In vitro experiments confirmed that cereulide induced ER stress by accumulating ROS. In addition, this study suggested that the treatment with sodium butyrate (NaB) inhibited cereulide-induced ER stress and alleviated apoptosis in liver and kidney tissues.

## 2. Results

### 2.1. Cereulide Exposure Induces Oxidative Stress and Inflammation in Mice

**Figure 1:**
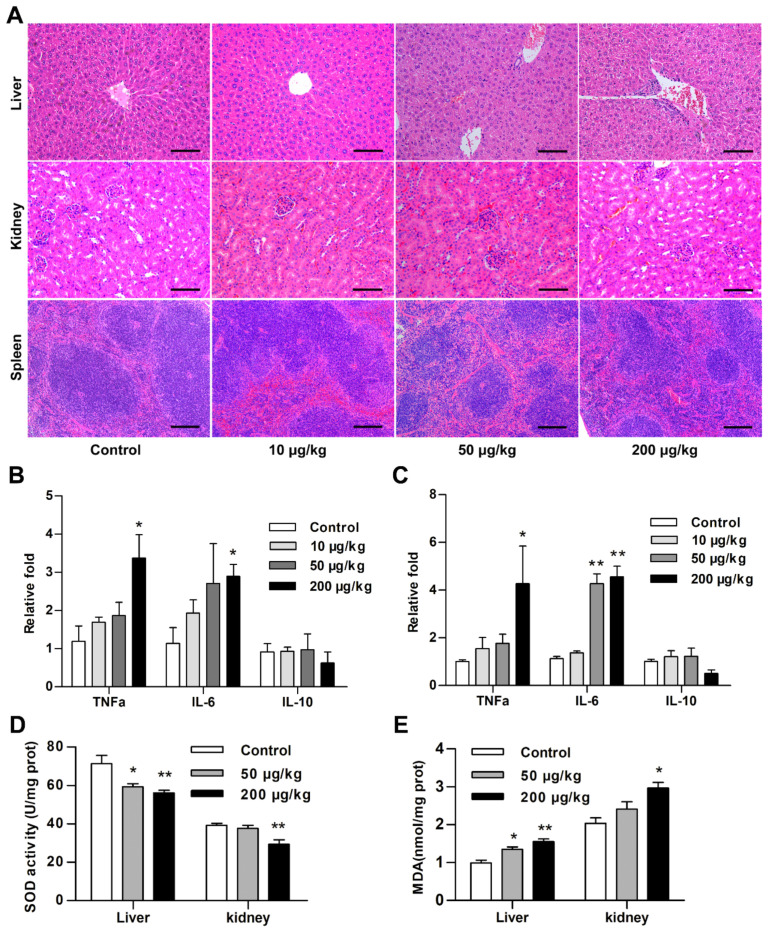
Cereulide exposure induces the oxidative stress production and the expression alteration of inflammatory cytokine. (**A**) Representative images of mouse liver, kidney and spleen tissues stained with H&E. Image magnification: ×200; scale bar = 100 µm. (**B**,**C**) The mRNA levels of *TNF-α*, *IL-6* and *IL-10* were detected in liver and kidney tissues by RT-qPCR. Data are shown as the mean ± SD (*n* = 5). (**D**,**E**) SOD activity and MDA levels in liver and kidney tissues exposed to different concentrations of cereulide (*n* = 5). For all panels, * *p* < 0.05, ** *p* < 0.01 compared to the control group.

Mice were administrated with cereulide by gavage respectively at 10, 50, and 200 μg/kg body weight for 28 days. No vomiting incidents were observed, and no abnormalities in their fur and mental state as well. However, the weight gain showed a downward trend in the 50 and 200 μg/kg group. The body weights in the 50 and 200 μg/kg group were significantly decreased than the control group from day 18 during 28 days of cereulide exposure. No significant difference in body weight of mice between 10 μg/kg group and control group was observed (Figure S1). In addition, the liver index in the 200 μg/kg group was significantly decreased, but the kidney index and spleen index weight were significantly increased (Table S2). The histopathology valuation by H&E staining showed that no apparent pathological changes were occurred in the liver, kidney, and spleen tissue of 10 μg/kg group, which indicated that 10 μg/kg of cereulide was the tolerable daily intake dose in mice. In contrast, the swelling of hepatocytes and glomeruli, inflammatory cell infiltration and white pulp lymphocyte reduction were observed in both 50 and 200 μg/kg group (Figure 1A). This suggested that relatively high dosage of cereulide (50 and 200 μg/kg) induced the inflammation response in the liver and kidney of mice during the long-time exposure. It was further confirmed by the analysis of the expression levels of some cytokines in the liver and kidney tissues that the mRNA levels of *IL-6* and *TNF-α* were increased in the liver and kidney with the cereulide exposure (50 and 200 μg/kg). The level of *IL-10* mRNA was downregulated at 200 μg/kg, but the difference was not statistically significant (Figure 1B,C).

Superoxide dismutase (SOD) activity and malondialdehyde (MDA) level as two important indicators were widely assayed for the occurrence of oxidative stress, because SOD eliminated superoxide anion and protect cells from oxidative stress, and MDA is an important product of lipid peroxidation. Compared with the control samples, the SOD activities were significantly reduced in the liver and kidney tissues of mice which are exposed to 50 and 200 μg/kg of cereulide, while the MDA levels were significantly increased in both liver and kidney tissues at 200 μg/kg exposure (Figure 1D,E). The decrease of SOD activity and the increase of MDA production showed as a dose dependent manner in the treated groups with 50 and 200 μg/kg cereulide for 28 days exposure, suggesting the correlation between the dose of cereulide exposure and the extent of oxidative stress. In brief, 10 μg/kg of cereulide seems a tolerable dose in mice, but 50 and 200 μg/kg of cereulide exposure induced oxidative stress and inflammation in mice.

### 2.2. Cereulide Exposure Triggers ER Stress and Induces Apoptosis of Liver and Kidney Cells in Mice

**Figure 2:**
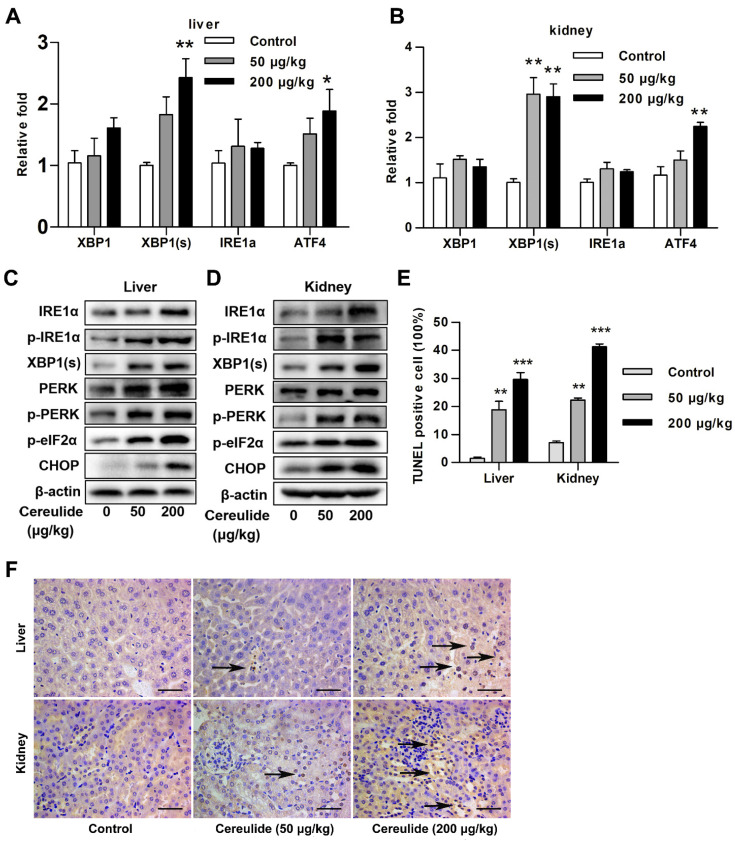
Long-time exposure to cereulide triggers ER stress and induces apoptosis of liver and kidney cells in mice. (**A**,**B**) The mRNA levels of *XBP1*, *XBP1s*, *IRE1α*, *ATF4* and *BiP* were detected in liver and kidney tissues by RT-qPCR. Data are shown as the mean ± SD (*n* = 5). (**C**,**D**) Western blot analysis for IRE1α, p-IRE1α, XBP1s, PERK, p-PERK, p-eIF2α, and CHOP in the liver and kidney tissues of mice exposed to cereulide (50 and 200 μg/kg). (**E**,**F**) TUNEL analysis of apoptosis level in liver and kidney tissue of mice exposed to cereulide (200 μg/kg). Arrows indicated apoptotic cells. Image magnification: ×400; scale bar = 50 µm. Data are presented as the mean ± SD (*n* = 6). For all panels, * *p* < 0.05, ** *p* < 0.01 and *** *p* < 0.001 compared to the control group.

Based on the results that cereulide exposure induced severe oxidative stress in the liver and kidney of mice, we addressed if cereulide induced ER stress in liver and kidney tissues, because oxidative stress is one of the important causes of ER stress. Considering that the pathological changes are not obvious in the 10 μg/kg group, we focused on the effect of cereulide exposure at 50 and 200 μg/kg in mice. Administration of cereulide increased the mRNA levels of *XBP1s* and activating transcription factor 4 (*ATF4*) in liver and kidney tissues with a dose-response relationship (Figure 2A,B). The protein or phosphorylation levels of IRE1α/XBP1(s) and PERK/eIF2α/CHOP were also increased at 50 and 200 μg/kg of cereulide exposure (Figure 2C,D). CHOP is a transcription factor that mediates apoptosis induced by ER stress. Therefore, the degree of apoptosis of liver and kidney tissues is detected by TUNEL assay. The results showed that the number of apoptotic cells was significantly increased when mice exposed to cereulide at 50 and 200 μg/kg. It is worth noting that apoptotic cells are mainly distributed around the central vein and renal tubules (Figure 2E,F). The animal experiments suggested that cereulide exposure induces ER stress, which further leads to apoptosis.

### 2.3. Cereulide Triggers Apoptosis by Activating ER Stress in HEK293T Cells and HepaRG Cells

**Figure 3:**
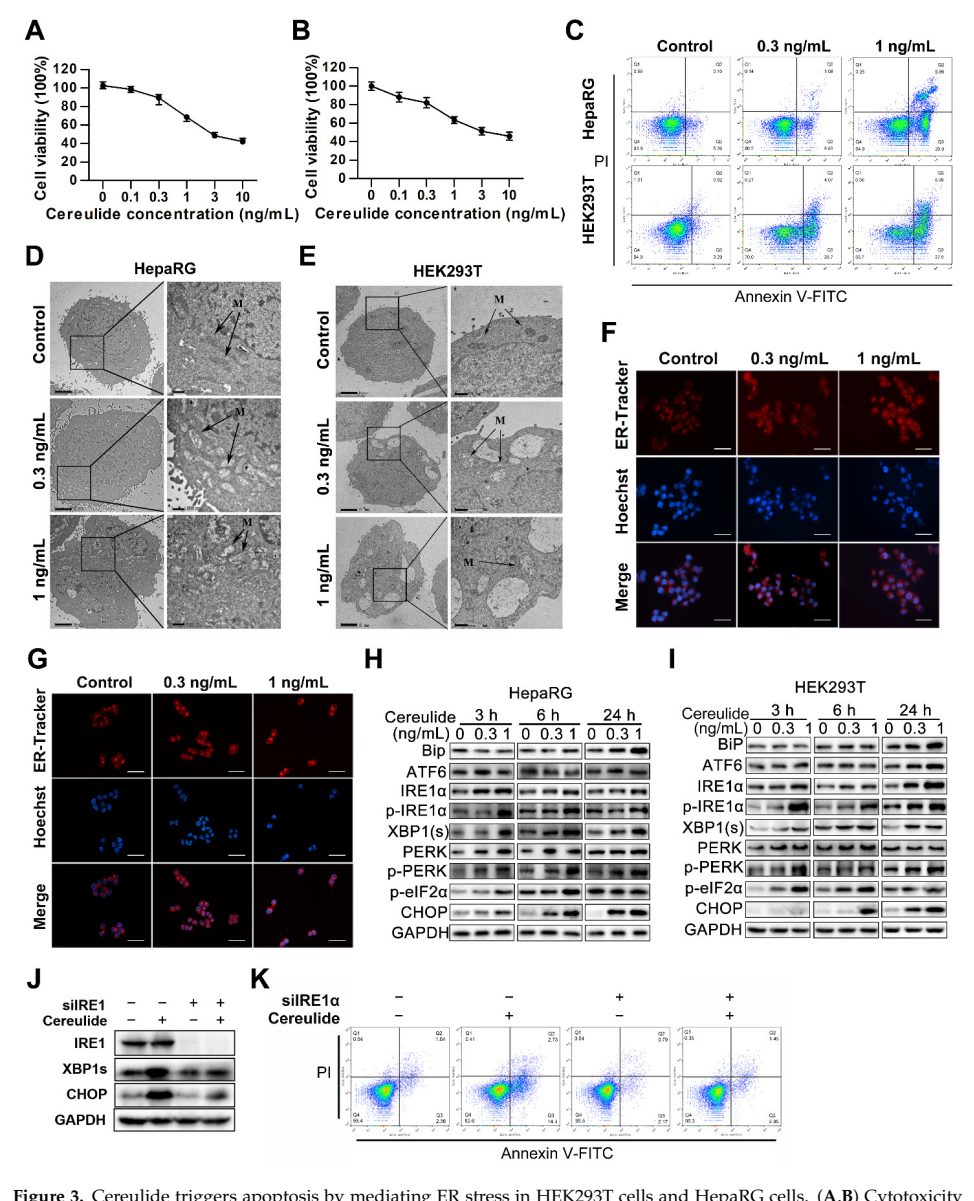
Cereulide triggers apoptosis by mediating ER stress in HEK293T cells and HepaRG cells. (**A**,**B**) Cytotoxicity of cereulide in HepaRG and HEK293T cells. HepaRG and HEK293T cells were treated with different concentrations of cereulide for 24 h. Cell viability was then determined with CCK-8 (*n* = 5). (**C**) Cell apoptosis resulting from cereulide treatment in HepaRG and HEK293T cells. HepaRG and HEK293T cells were evaluated with 0.3 and 1 ng/mL cereulide by flow cytometry. The data were analyzed using FlowJo software (*n* = 3). (**D**,**E**) Representative transmission electron microscopy images of HepaRG and HEK293T cells treated with cereulide toxin. Cereulide treated HepaRG and HEK293T cells for 24 h, and observed by transmission electron microscope. M indicates mitochondria. Scale bars: left panel, 2 µm; right panel, 500 nm. (**F**,**G**) ER labeling. HepaRG and HEK293T cells were treated with 0, 0.3, 1 ng/mL cereulide for 24 h. Image magnification: ×200; scale bar = 100 µm. The cells were stained with ER-Tracker and observed under a fluorescence microscope at 587 nm excitation and 615 nm emission. (**H**,**I**) ER stress-related protein levels during cereulide exposure were evaluated in HepaRG and HEK293T cells. HepaRG and HEK293T cells were exposed to cereulide toxin (0, 0.3, 1 ng/mL) for 3, 6 and 24 h and Western blot analysis for BiP, ATF6, IRE1α, p-IRE1α, XBP1(s), PERK, p-PERK, p-eIF2α, CHOP. (**J**) IRE1α knock-down blocked cereulide to down-regulate XBP1s. HepaRG cells were transfected with scramble siRNA as negative control (NC) or IRE1α siRNA for 24 h and then exposed to cereulide (1 ng/mL) for 6 h. (**K**) IRE1α knock-down blocked cereulide to increase in apoptosis. HepaRG cells were transfected with scramble siRNA as negative control (NC) or IRE1α siRNA for 24 h and then evaluated with 1 ng/mL cereulide for 24 h by flow cytometry (*n* = 3).

To further explore the toxicological mechanism of cereulide in liver and kidney, HepaRG cells and HEK293T cells were chosen to be treated with different concentrations of cereulide for 24 h, and the cell viabilities were determined by the CCK8 method. The IC50 value for HepaRG cells was 2.98 ng/mL of cereulide, and the IC50 value for HEK293T cells was 2.74 ng/mL (Figure 3A,B). According to that, 0, 0.3, and 1 ng/mL of cereulide were applied in the treatment of HepaRG cells and HEK293T cells in the follow-up studies. The results of flow cytometry showed the number of apoptotic cells was significantly increased exposed to cereulide for 24 h, which was dose-dependent (Figure 3C). TEM (transmission electron microscopy) results showed that mitochondria were swollen and ruptured, and obvious apoptosis phenomena such as cell membrane invagination, chromosome condensation, and autophagosomes appeared in HepaRG and HEK293T cells (Figure 3D,E).

We have found that cereulide induced ER stress in animal models. Next, we tested this finding in HEK293T cells and HepaRG cells to explore the underlying toxicological mechanism. Firstly, ER was stained with ER-tracker, a red fluorescent probe specifically dying ER, and showed that the ER was swollen in both 0.3 ng/mL and 1 ng/mL of cereulide administrations (Figure 3F,G). Second, we found that cereulide can cause ER stress to upregulate the expression of the pro-apoptotic transcription factor CHOP, which is obviously time- and dose-dependent (Figure 3H,I). Besides of that, with the increase of time and concentrations in cereulide treatment, the phosphorylation levels of IRE1α and PERK were increased significantly, suggesting that IRE1α and PERK were activated, which consequently promoted the transcription factor XBP1 splicing and increased the phosphorylation level of eIF2α (Figure 3H,I). No apparent increase of BiP level was observed at the exposure time 3 h and 6 h, but BiP significantly increased at 24 h exposure (Figure 3H,I). All these data showed that cereulide stimulation triggered ER stress in HepaRG and HEK293T cells. In HepaRG cells, IRE1α knockdown reduced the upregulations of XBP1s and CHOP expression caused by cereulide exposure (Figure 3J), and alleviated cereulide-induced apoptosis (Figure 3K). These data indicated the critical role of ER stress in cereulide-induced toxicosis.

### 2.4. Cereulide Exposure Induces Oxidative Stress and Inflammatory Cytokine Production In Vitro

**Figure 4:**
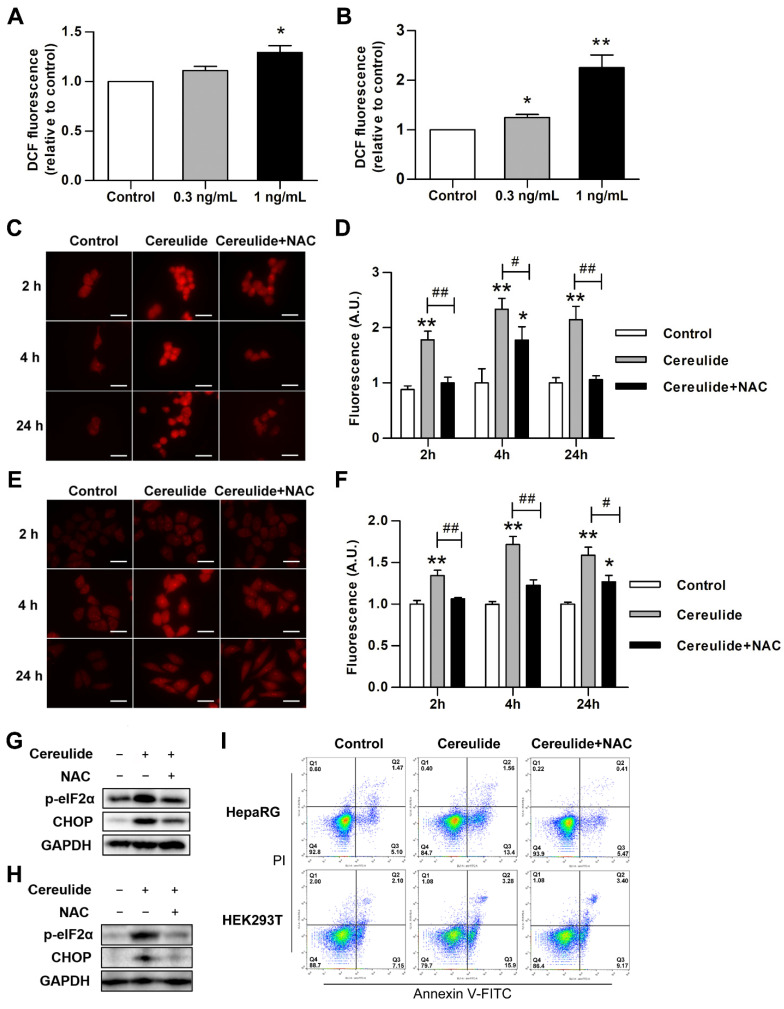
Cereulide induces ER Stress response by ROS production. (**A**,**B**) Cereulide causes an increase in ROS production. HepaRG and HEK293T cells were treated with 0, 0.3, 1 ng/mL cereulide for 24 h, the fluorescence of dichlorofluorescindiacetate (DCF) were detected at 488 nm excitation and 525 nm emission; *n* = 5. (**C**,**D**) Effects of ROS inhibitor on ROS production after cereulide treatment. HEK293T cells were pretreated with 5 mM NAC followed by treatment of 0.5 ng/mL cereulide for 2, 4, 24 h. Cytosolic ROS production measured by DHE labeling, *n* = 5. (**E**,**F**) HepaRG cells were pretreated with 7 mM NAC followed by treatment of 1 ng/mL cereulide for 2, 4, 24 h; *n* = 5. For C and E panels, image magnification: ×400; scale bar = 50 µm. (**G**,**H**) ROS inhibitor relieves ER stress caused by cereulide. According to the dosage described above, HepaRG and HEK293T cells were treated for 4 h and Western blot analysis for p-eIF2α, CHOP. (**I**) NAC treatment reduce the apoptosis caused by cereulide. HepaRG and HEK293T cells were treated as described above and then evaluated by flow cytometry; *n* = 3. For all panels, * *p* < 0.05 and ** *p* < 0.01 compared to the control cells; # *p* < 0.05 and ## *p* < 0.01 compared to NAC treated cells.

We observed that cereulide triggers apoptosis by mediating ER stress and damages mitochondria. Mitochondria are the main place for ROS production. The ROS levels in HepaRG and HEK293T cells treated by cereulide, were significantly increased in a dose-dependent manner (Figure 4A,B). This result is consistent with the occurrence of oxidative stress in liver and kidney tissues in animal experiments. To further determine whether ROS production plays an important role in the process of cereulide-induced ER stress, we used ROS inhibitor N-Acetyl-L-cysteine (NAC) for intervention. Compared with the cell treated with cereulide, adding of NAC in cereulide administrated cells significantly alleviated the increase in ROS production at different treatment time (Figure 4C–F). In Figure 3, it was already demonstrated that ER stress was apparently activated after exposure to cereulide for 3 to 6 h. Therefore, we detected changes of two ER stress marker, the protein amount of CHOP and phosphorylation level of eIF2α at 4 h of combined treatment with NAC and cereulide. The results showed that NAC treatment effectively reversed the increases of p-eIF2α and CHOP caused by cereulide in both HepaRG and HEK293T cells (Figure 4G,H). As expected, NAC treatment significantly reduced the extent of apoptosis caused by cereulide administration (Figure 4I). Taken together, the overproduction of ROS is the main factor for the activation of ER stress under cereulide exposure.

### 2.5. NaB Relieves Cereulide-Induced Liver and Kidney Damages by Inhibiting ER Stress

**Figure 5:**
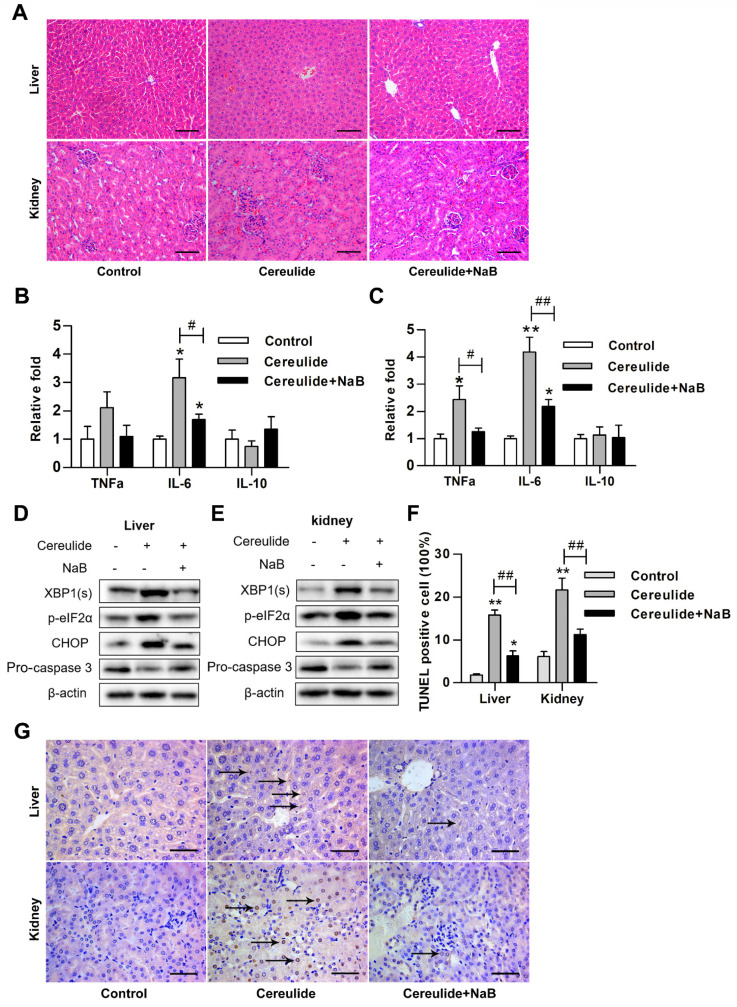
NaB relieves cereulide-induced liver and kidney damage by inhibiting ER stress. (**A**) Representative images of H&E-stained liver and kidney tissues from cereulide + NaB-treated mice and cereulide-treated mice (×200 magnification). (**B**,**C**) The mRNA levels of TNF-α, IL-6 and IL-10 were detected in liver and kidney tissues by RT-qPCR, *n* = 5. (**D**,**E**) Western blot analysis for XBP1(s), p-eIF2α, CHOP and Caspase3 in the liver and kidney tissues of mice that exposed to cereulide (50 μg/kg) were treated with NaB (100 mg/kg) for 28 days. (**F**,**G**) TUNEL analysis of apoptosis level in liver and kidney tissue of mice exposed to cereulide (50 μg/kg) and cereulide + NaB (100 mg/kg) (×400 magnification). Arrows indicated apoptotic cells. Data are presented as the mean ± SD (*n* = 6). For all panels, * *p* < 0.05 and ** *p* < 0.01 compared to the control group; # *p* < 0.05 and ## *p* < 0.01 compared to cereulide + NaB group.

The results have shown that cereulide promoted ROS production, activated ER stress, and caused apoptosis step-wisely. Activation of the IRE1α/XBP1 and PERK/eIF2α pathways seems to play the central role in cereulide toxicosis due to its linkages with apoptosis and inflammation. Our previous study has proven that NaB supplementation effectively inhibited the activation of ER stress caused by deoxynivalenol [17]. We also observed in HepaRG and HEK293T cells that NaB inhibits the activation of IRE1α/XBP1 pathway and reduces the up-regulation of CHOP expression caused by cereulide (Figure S2). In addition, butyrate has an anti-inflammatory effect [18]. It is interesting to examine the protective effect of NaB in mice on defending cereulide-induced apoptosis and inflammation via inhibiting ER stress. The histological analysis showed that NaB treatment effectively attenuates hepatocyte vacuolar degeneration and glomerular swelling caused by cereulide (Figure 5A). Supplementation of NaB apparently inhibits the up-regulation of pro-inflammatory factors *IL-6* and *TNFα* mRNA levels which was occurred in cereulide administration (Figure 5B,C). Cereulide-induced ER stress was also reduced, referred to decrease of the levels of XBP1(s) expression, eIF2α phosphorylation, CHOP expression and caspase3 cleavage (Figure 5D,E). As a consequence, NaB supplementation significantly inhibited the apoptosis of liver and kidney tissues caused by cereulide (Figure 5F,G). Together, these results suggested that NaB treatment blocked ER stress and inhibited the liver and kidney damages induced by cereulide.

## 3. Discussion

Plenty reports evidenced that cereulide contamination exists in common daily foods. As reported in the European Union (EU), every year, 500 to 700 confirmed human cases of foodborne diseases were caused by *B. cereus*, and the incidence is annually increased [19]. In the Dutch market, 0.7% of the food samples contained *B. cereus* exceeding the EU pollution standards, 16.8% of tested isolates either produced cereulide or carried the ces gene, and the cereulide content of the two samples reached 3.2 and 5.4 μg/kg [20]. Cereulide-producing *B. cereus* has been isolated from baby food and re-inoculated into food at room temperature for 24 h to accumulate 2–200 μg cereulide per 100 mL of food [21]. *B. cereus* strains are commonly detected in pasteurized milk, and some of them produce cereulide [22,23].

There are a wide range of foods contaminated by cereulide, and cereulide derived from different foods is easily cross-enriched in the body, so that the body is possibly exposed at a sub-emetic concentration of cereulide situation for a long time. In addition, sub-emetic cereulide probably aggravates the toxicological effects caused by other pathogenic factors such as mycotoxins in intestinal barrier dysfunction [24]. The current animal toxicology models of cereulide mainly explore the acute toxic effects through a single exposure [7,25,26]. The continuous exposure to cereulide based on cell lines lacks the body’s internal barriers and is not able to accurately reflect the overall impact of cereulide on the individual level. Our study explored the toxicological mechanism of cereulide to the body by continuously exposing different doses, 10, 50, and 200 μg/kg bw of cereulide to mice. Results showed 10 μg/kg of cereulide exposure did not exhibited obvious toxic effects in mice. Previous study also found that piglets taken in the 7 days exposure were not significantly affected by daily administration of 10 μg/kg bw cereulide [7]. It is interesting that neither emesis nor death has been observed in 50 and 200 μg/kg bw of cereulide treated groups for 28 days exposure, but both administrations induced oxidative stress and inflammation in the liver and kidney of mice. However, it has been estimated that the amount of cereulide was ranged from 80 to 150 μg/kg bw in the food outbreak by the researchers’ calculation [5,6]. This discrepancy might reflect that BALB/c mice possibly have much higher tolerance to cereulide exposure than human does. Or perhaps, in addition to cereulide producing, *B. cereus* also possibly produced other toxins such as hemolysin BL, nonhemolytic enterotoxin, cytotoxin K and phospholipase, etc, which auxiliary contributed to toxic effects in human body [27]. Additionally, the slight pathological changes of liver were caused by a single intraperitoneal injection of 5 μg cereulide per mouse [25]. This was comparable with 200 μg/kg bw exposure (average body weight as 22.3 g in the study). Herein, we confirmed that the long-time exposure of cereulide with high dose caused obvious damages of liver and kidney in mice. Besides that, our recent study also found that cereulide exposure caused intestinal inflammation and gut microbiota dysbiosis in mice [28]. The combined application of cereulide and DON can promote intestinal inflammation [16]. All these finding suggested that cereulide exposure could induced multiple organ injuries at high concentrations.

Our study on liver and kidney tissues found that cereulide exposure activates ER stress and stimulates the increase of inflammatory cells. In vitro and in vivo results showed that cereulide indeed induces ER stress and mediates the apoptosis of liver and kidney through the IRE1α/XBP1 and PERK/eIF2α pathways (Figure 2 and Figure 3). Similarly, our previous studies found that cereulide exposure also activated ER stress IRE1/XBP1/CHOP pathway to induce cell apoptosis and inflammatory cytokines production in mouse intestine [28]. It is contrary to previous study stated that cereulide only activated ATF4 and CHOP pathways, but not phosphorylated the upstream activator PERK, so it is not caused by activation of ER stress [15]. The possible reasons for the discrepancy might result from the differences in the treatment time and cell lines. Our results showed that cereulide causes mitochondrial swelling, in agreement with the evidence that cereulide causes mitochondrial dysfunction [29]. Since mitochondria are the main site of ROS production, the ROS produced by oxidative stress affected the function of disulfide bonds and cause protein misfolding in the endoplasmic reticulum [30,31]. It is reasonable that NAC, the ROS inhibitor, reduced the activation of PERK-eIF2α-CHOP axis and prevented cell from apoptosis. This indicated that cereulide exposure induces ROS accumulation to cause ER stress, and consequently, the UPR pathway is unbalanced and leads to apoptosis.

After exposed to cereulide, the expression of IRE1α was increased significantly in mouse liver and kidney tissues, as well in HepaRG and HEK293T cells. Upregulation of IRE1α promotes the production of inflammatory cytokines by restricting endonuclease activity and regulating transcription [32,33]. In this study, we found that IRE1 plays a dual role in inflammation and ER stress-mediated apoptosis caused by cereulide. NaB has a significant inhibitory effect on the IRE1/XBP1 and PERK/CHOP pathways [34,35]. In addition, butyrate exerts anti-inflammatory effects by inhibiting the activation of NF-κB and IFN-γ signaling [36,37]. In agreement with these studies, NaB supplementation efficiently inhibited the activation of ER stress, reduced the transcription of pro-inflammatory factors, and partially antagonized the apoptosis of liver and kidney cells. NaB has multiple effects such as enhancing the intestinal barrier and mucosal immunity [38]. Therefore, it is speculated that the antagonism of NaB on the toxicosis of cereulide may be achieved by repairing the damaged intestinal barrier, reducing inflammation during the early absorption, or inhibiting intake of cereulide in mice. This speculation needs further verifications especially in the intestine. In general, NaB has a potential as a therapeutic strategy for cereulide exposure.

## 4. Materials and Methods

### 4.1. Chemicals and Reagents

Cereulide powder was purchased from Chiralix (Nijmegen, The Netherlands) with a purity of 99.0% and dissolved in 20% ethanol. N-Acetyl-L-cysteine (NAC, purity of ≥99%) was purchased from Sigma-Aldrich (St. Louis, MO, USA). Sodium butyrate (NaB, purity of ≥98%) was purchased from Sangon Biotech (Shanghai, China). Antibodies against CHOP (AC532-L63F7), p-eIF2α (AF1237), XBP1s (AF8366), Phospho-PERK (AF5902) was purchased from Beyotime Biotech (Shanghai, China), anti-ATF6 (D262665) were purchased from BBI (Shanghai, China). Antibodies against BiP (ab108615-EPR4041(2)) was purchased from Abcam (Cambridge, UK). Antibodies against Caspase3 (#9662), PERK (#5683-D11A8), IRE1α (#3294), β-Actin (#4970-13E5) and anti-rabbit HRP-linked Antibody (#7074) and anti-mouse HRP-linked antibody (#7076) were purchased from Cell Signaling Technology (Danvers, MA, USA). Antibodies against p-IRE1α (#13013) was purchased from Signalway Antibody (College Park, MD, USA). Antibodies against GAPDH (sc47724-0411) was purchased from Santa Cruz Biotechnology (Santa Cruz, CA, USA).

### 4.2. Experimental Animals

Five-week-old specific pathogen-free (SPF) male BALB/c mice were purchased from Guangdong Medical Laboratory Animal Center (GDMLAC, Foshan, China) and housed under standard conditions of temperature (23 ± 2 °C), humidity (40–60%), and kept under a 12 h/12 h light–dark cycle. All animal experiments were approved by the Institutional Animal Care Committee of South China Agricultural University (Permit NO. 2020b068, Date: 15 September 2020) and performed in accordance with the approved relevant guidelines. The mice were acclimated to the environment for one week and then randomly divided into a control group, 10 μg/kg bw group, 50 μg/kg bw group and 200 μg/kg bw group, with six mice in each group. The control group was given 0.2 mL of 10% ethanol daily by gavage; the other three groups were challenged orally cereulide diluted with 10% ethanol at the concentration of 10, 50, 200 μg/kg body weight (200 μL) respectively. The dosage was chosen based on previous work [25]. In the research on the protective effect of NaB on cereulide-exposure mice, BALB/c mice were randomly divided into three groups (*n* = 5) and challenged orally following treatments: 10% ethanol (Control), 50 μg/kg cereulide and 100 mg/kg NaB + 50 μg/kg cereulide (Cereulide + NaB). The dosage of NaB in the Cereulide + NaB group is chosen according to previous research [34].

All the administrations were conducted for 28 days, and the weights and mental state of the mice were recorded. At the end of the experiment, the mice were killed by cervical dislocation. Liver, kidney, and spleen of mice were collected, some were fixed with 4% paraformaldehyde, and some were frozen at −80 °C.

### 4.3. Cell Culture

The human hepatocarcinoma cell line (HepaRG) was purchased from Shanghai Guandao Biological Engineering Co., Ltd. (Shanghai, China) and cultured in RPMI Medium 1640 supplemented with 10% fetal bovine serum (FBS; PAN-Biotech, Aidenbach, Germany). The human embryonic kidney cell line 293T (HEK293T) was purchased from American Type Culture Collection (ATCC; Manassas, VA, USA) and cultured in Dulbecco’s Modified Eagle Medium (DMEM; Gibco, Waltham, MA, USA) supplemented with 10% FBS. HepaRG cells and HEK293T cells were cultured in a 5% carbon dioxide atmosphere at 37 °C in a constant-temperature incubator.

### 4.4. CCK-8 Cell Viability Assay

The cell viability was detected by Cell Counting Kit-8 (CCK8) based on 2-(2-Methoxy-4-nitrophenyl)-3-(4-nitrophenyl)-5-(2,4-disulfophenyl)-2H-tetrazolium Sodium Salt (WST-8) reaction principle (Yeasen, Shanghai, China). Briefly, the cells were seeded into 96-well plates and treated with different concentrations of cereulide toxin for 24 h. Then, the WST-8 reagent was added to each well for incubation at 37 °C for 2 h. The samples were quantified by measuring the absorbance of the formazan at 450 nm with an ELISA plate reader. Refer to IC50 value of cells in this study, HepaRG and HEK293T cells were treated with 0.3 and 1 ng/mL cereulide toxin in the following experiments.

### 4.5. Plasmid Transfection and Small Interfering RNA (siRNA) Analysis

HepaRG cells and HEK293T cells were transfected with the IRE1α siRNA, PERK siRNA (100 nM) and negative control (NC) siRNA as previously described [19]. The IRE1α siRNA sequence (GGACGUGAGCGACAGAAUAdTdT), the PERK siRNA sequence (GUGACGAAAUGGAACAAGATT) and NC sequence (UUCUCCGAACGUGUCACGUTT) were synthesized by Gene Pharma (Shanghai, China).

### 4.6. Determination of Apoptotic Cells

Quantitation of the apoptotic cells treated with cereulide and without cereulide was obtained using the Annexin V-FITC detection kit (Beyotime Biotech, Shanghai, China). The cells were collected and stained with Annexin V-FITC and propidium iodide according to the manufacturer’s instructions, and apoptotic cells were measured by Fluorescence activated Cell Sorting (FACS) analysis.

### 4.7. Electron Microscopy

The cultured cells were prefixed in 2.5% glutaraldehyde overnight at 4 °C, washed in PBS buffer and then postfixed in 1% osmium tetroxide for 2 h. The cells were dehydrated in different concentrations of ethanol and acetone, and infiltrated with neutral resin. Ultrathin sections (70 nm) were stained with uranyl acetate and lead citrate at room temperature and then observed under a transmission electron microscope (FEI, Eindhoven, The Netherlands).

### 4.8. Reactive Oxygen Species (ROS) Measurement

After cereulide treatment, cells were washed and then incubated with either the oxidative fluorescent probe dichlorofluorescein-diacetate (DCFH-DA; Beyotime Biotech, Shanghai, China) for 30 min at 37 °C or Dihydroethidium (DHE; Yeasen, Shanghai, China) for 20 min at 37 °C. The results were observed using fluorescence microscopy (Zeiss, Oberkochen, Germany) or measured at 488 nm excitation and 525 nm emission by Multi-Mode microplate reader (Molecular Devices, San Jose, CA, USA).

### 4.9. Western Blotting

Total protein of cells and tissues were extracted using RIPA lysis buffer containing 1 mM PMSF (Beyotime Biotech, Shanghai, China). The protein was separated by sodium dodecyl sulfate poly-acrylamide gel electrophoresis (SDS-PAGE) and transferred to polyvinylidene difluoride (PVDF) membranes (Millipore, Burlington, MA, USA). Non-specific binding sites were blocked with 5% nonfat milk in TBST and the membranes were incubated with specific primary antibodies against CHOP, p-eIF2α, IRE1α, XBP1s, p-PERK, ATF6, BiP, and Caspase3, p-IRE1α, GAPDH, PERK, β-Actin. The membranes were then incubated with anti-rabbit or anti-mouse HRP-linked antibody. Protein bands were detected by ChemiDocXRS system (Bio-Rad, Hercules, CA, USA) using enhanced chemiluminescence reagents (Beyotime Biotech, Shanghai, China).

### 4.10. Malondialdehyde (MDA) and Superoxide Dismutase (SOD) Assays

The liver and kidney tissues were ground into powder with liquid nitrogen, then fully lysed with cell lysis buffer (Beyotime Biotech, Shanghai, China) on ice, tissue lysates were centrifuged at 12,000 g for 5 min at 4 °C to collect the supernatant. Malondialdehyde (MDA) levels were measured using the Lipid Peroxidation MDA Assay Kit (Beyotime Biotech, Shanghai, China) according to the instruction of manufacturer. Superoxide Dismutase (SOD) activity were detected by the Toal SOD Assay Kit with WST-8 (Beyotime Biotech, Shanghai, China) and calculated according to the instruction of manufacturer.

### 4.11. Real-Time Quantitative PCR (QPCR)

Total RNA was isolated from liver and kidney tissues of mice with the TRIZOL reagent (Invitrogen, Waltham, MA, USA), and then performed reverse transcription using the ReverTra Ace QPCR RT Kit (TOYOBO, Osaka, Japan), according to the manufacturer’s recommendations. cDNA samples and specific primers (Supplementary Table S1) were used to amplify the target genes using Hieff Qpcr SYBR Green Master Mix (Yeasen, Shanghai, China) on Bio-Rad Opticon system. The gene expression analysis was normalized to the expression of GAPDH. Relative fold was calculated using the 2^−ΔΔCt^ method.

### 4.12. Histopathology and TUNEL Analysis

Liver and kidney tissues were fixed in 10% neutral formalin and embedded in paraffin. Then, 5 μm-thick sections were stained with hematoxylin and eosin (H&E). The results were examined with light microscopy (Leica Microsystems, Heidelberg, Germany). Furthermore, tissue slides were pretreated with proteinase K and H_2_O_2_, followed by incubation with TUNEL reaction mixture at 37 °C for 60 min using DAB (SA-HRP) TUNEL Cell Apoptosis Detection Kit (Servicebio, Wuhan, China) following the manufacturer’s guidelines. The percentage of apoptotic cells was calculated by dividing the number of TUNEL-positive cells by the total number of cells visualized in the same field.

### 4.13. Statistical Analysis

Data were expressed as the mean ± SD. All statistical analyses were performed using SPSS 21.0 software (IBM, Armonk, NY, USA). Statistical differences among different groups were evaluated by one-way analysis of variance (ANOVA) with the least significant difference (LSD) test. Statistical differences were considered significant at a *p* value of < 0.05.

## 5. Conclusions

Long-time exposure to cereulide would induce inflammation and cause the liver and kidney apoptosis, which led to an increased risk of liver atrophy and chronic kidney disease. Mechanistically, we found that cereulide-induced apoptosis is triggered by ER stress, and ROS accumulation triggers ER stress. Supplementation of NaB effectively protected the liver and kidney damages caused by cereulide through inhibiting the ER stress pathway. Our research provides a new perspective on the toxicological mechanism and prevention of cereulide induced toxicosis.

## Data Availability

Data is contained within the article and supplementary material.

## Supplementary Materials

The following are available online at https://www.mdpi.com/article/10.3390/ijms22179148/s1.
