# Supplementary material for: Cereulide Exposure Caused Cytopathogenic Damages of Liver and Kidney in Mice
Source: Int J Mol Sci. 2021 Aug 24;22(17):9148. doi: 10.3390/ijms22179148 (PMC8431326; doi:10.3390/ijms22179148)
Supplement: Supplementary file 1 [file ijms-22-09148-s001.zip › ijms-1295532-SI.pdf]

Table S1 Primer sequences used for RT-qPCR analysis (F: forward; R: reverse)

Group	Liver weight /Body weight (%)	Kidney weight /Body weight (%)	Spleen weight /Body weight (‰)
Control	4.17±0.068	1.58±0.038	2.95±0.17
200 μg/kg Cereulide	3.95±0.063*	1.65±0.038*	3.68±0.25*
50 μg/kg Cereulide	4.00±0.046	1.66±0.018*	3.34±0.22
10 μg/kg Cereulide	3.99±0.022	1.63±0.042	3.21±0.086

Table S2 Effect of Cereulide Exposure on Organ index in Mice

Primer name	Primer sequence (5′-3′)
Mouse IL-6	F: ACCTGTCTATACCACTTCACAAGT
	R: TCTGCAAGTGCATCATCGTTGTTC
Mouse TNF-α	F: CCAGACCCTCACACTCAGATC
	R: AGTTGGTTGTCTTTGAGATCCATG
Mouse IL-10	F: GGTTGCCAAGCCTTATCGGA
	R: ACCTGCTCCACTGCCTTGCT
Mouse GAPDH	F: GAGAAACCTGCCAAGTATGATGAC
	R: TAGCCGTATTCATTGTCATACCAG
Mouse BIP	F: CGACCTGGGGACCACCTACT
	R: TTGGAGGTGAGCTGGTTCTT
Mouse ATF4	F: CATGGCGTATTAGAGGCAGC
	R: ACACTGCTGCTGGATTTCGT
Mouse Xbp1(s)	F: ACACGCTTGGGAATGGACAC
	R: CCATGGGAAGATGTTCTGGG
Mouse Xbp1(u)	F: ACACGCTTGGGAATGGACAC
	R: GAGTTTTCTCCCGTAAAAGCTGA
Mouse IRE1α	F: GCCCCGGGAGTTTTGG
	R: GGGTCGAGACAAACAACAAGGT

Data are shown as the mean ± SD (n = 5), * $p < 0.05$ when compared to the control group.

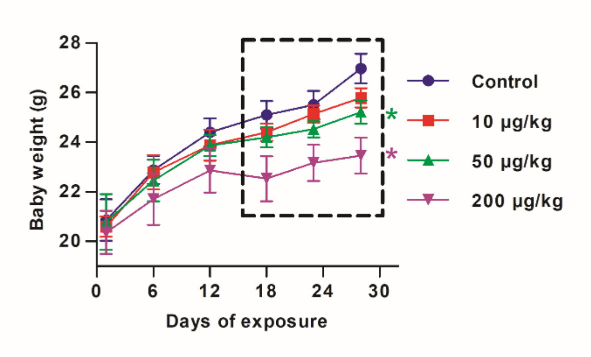

Figure S1 Effect of cereulide exposure on the body weight in mice

Mice were exposed to 50 and 200 μg/kg of cereulide for 28 days, and their body weights were significantly lower than that of the control group from day 18th to day 28th. * $p < 0.05$ compared to the control group.

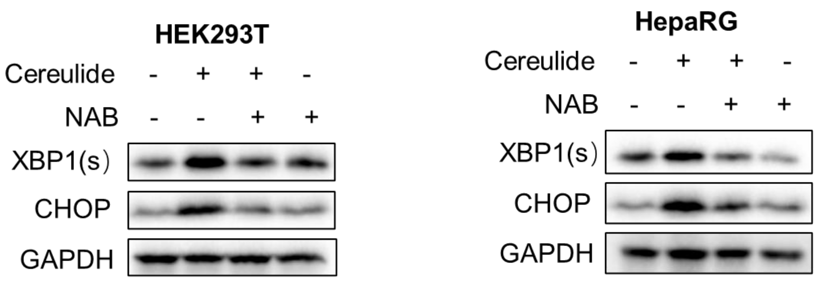

Figure S2 NaB inhibits ER stress caused by cereulide in HepaRG and HEK293T cells

Western blot analysis for p-eIF2α, XBP1(s) and CHOP in HepaRG and HEK293T cells that were untreated or treated with cereulide (0.3 ng/mL) with or without NaB (0.5 mM) for 48 h.
